# The Efficacy of Psychological Capital Intervention (PCI) for Depression From the Perspective of Positive Psychology: A Pilot Study

**DOI:** 10.3389/fpsyg.2019.01816

**Published:** 2019-08-07

**Authors:** Ruijun Song, Nana Sun, Xuhong Song

**Affiliations:** ^1^School of Politics and Law, Shaoyang University, Shaoyang, China; ^2^School of Psychology, Shandong Normal University, Jinan, China; ^3^Department of Education, Luliang University, Luliang, China; ^4^Department of Medical Psychology, Shanxi Medical University, Taiyuan, China

**Keywords:** depression, psychological capital, intervention, positive psychology, pilot study

## Abstract

Extensive psychological interventions primarily target the negative symptoms of depression and the deficits in positive resources have been systematically neglected. So far, little attention has been devoted to psychological capital (PsyCap) intervention from the perspective of developing positive resources. The aim of the present pilot study was to evaluate the efficacy of psychological capital intervention (PCI) for depression in a randomized controlled trial. A total of 56 patients were randomized to either care as usual (CAU) for normal medication or psychological capital intervention (PCI) group, where the normal medication was supplemented with the PCI. Participants were assessed at pre- and post-treatment, as well as 6-month follow-up, on measures of depressive symptoms and PsyCap. The PCI group displayed significantly larger improvements in PsyCap and larger reductions in depression symptoms from pre- to post treatment compared to control group. Improvements were sustained over the 6-month follow-up period. Targeting the positive resources intervention in the PCI may be effective against the treatment of depression.

## Introduction

Depression is a highly prevalent mental disorder with lifetime prevalence rates for depression at over 16% (Kessler, 2003). It is recurrent, debilitating and even lethal. The onset of depression is accompanied by a host of undesirable health, social consequences, it also creates a substantial financial burden (Dunlop et al., 2005; Lopez and Mathers, 2006; Kessler, 2012; Papakostas and Ionescu, 2014). Given these ramifications, it is critical to concentrate on intervention efforts.

Currently, depression intervention to date has focused largely on fixing negative or pathological feelings, thoughts and behaviors (Sin and Lyubomirsky, 2009). Previous studies have examined the efficacy of psychological treatments for depression including cognitive-behavioral therapy (CBT), behavioral therapy, interpersonal therapy and problem solving therapy (Hollon and Ponniah, 2010; Cuijpers et al., 2011; Barth et al., 2016). There are no major differences in efficacy between these psychotherapies and all demonstrated their efficacy (Cuijpers et al., 2008).

However, for some depressed individuals, therapies that mainly focused on negative emotions and cognitive biases may be counterproductive to their recovery and may disrupt the therapeutic alliance (Burns and Nolen-Hoeksema, 1992; Castonguay et al., 2004). Thus, some researches elucidated that positive psychology interventions, which aims to cultivate positive resources (i.e., positive feelings, behaviors and cognitions) may serve as an important yet underexplored intervention approach in facilitating recovery from depression (Fredrickson, 2013; Layous et al., 2014; Taylor et al., 2017).

Evidence across multiple units of analysis reveals that depression is associated with absence of positive resources (Hofmann et al., 2012). Additionally, there is robust evidence that decreased positive resources are implicated in apathy, inattentiveness, lack of interest and lack of self-confidence and sadness, which are pivotal characteristics of depression (Watson et al., 1988; Clark and Watson, 1991). Recent studies have noted that positive resources can reduce the depressive symptoms and might play a protective role in the development of depression (Bos et al., 2013; Lindahl and Archer, 2013; Raes et al., 2014; Boumparis et al., 2016). The unique link between positive resources and depression suggests that intervention targeting the positive resources may fill a particularly important gap left by extant treatment. A typical example for positive resources is psychological capital. Psychological capital (PsyCap), describes an individual’s psychological capacity that can be developed, and managed for performance improvement (Luthans, 2002). Psychological capital is composed of: (1) hope: persevering toward goals and when necessary, redirecting paths to goals in order to succeed; (2) self-efficacy: having confidence to succeed a challenging tasks; (3) optimism: making a positive attribution regarding succeeding now and in the future; (4) resiliency: when beset by problems and adversity, sustaining and bouncing back and even beyond to attain success (Luthans and Youssef, 2004; Luthans et al., 2007a). Recent research demonstrated that PsyCap was significantly related to symptoms of depression (Bakker et al., 2017). In addition, evidences indicated that PsyCap can significantly moderated the associations of depression and stress, could be a positive resource for combating depression (Liu et al., 2012; Shen et al., 2014).

The PsyCap intervention (PCI) is a highly focused training session which draws from past research in terms of hope, self-efficacy, optimism and resilience (Luthans et al., 2007b). According to the resource activation theory, PCI concentrating on positive aspects of the person (i.e., resource priming), can promote positive perception, behavioral and emotion, which in turn enhance resource realization and lead to a positive mood (Grawe and Grawe-Gerber, 1999; Flückiger and Wüsten, 2009). There is accumulating evidence that PCI can drastically increase the individual’s level of PsyCap, well-being and organizational behaviors (Luthans et al., 2010; Avey et al., 2011; Newman et al., 2014; Nolzen, 2018). However, to our best of knowledge, no studies have examined the effect of PCI in the treatment of depression.

The central aim of the current pilot study was to test the efficacy of PCI in a sample of depressed individuals. We hypothesized that participants assigned the PCI group would report greater reductions of depression symptoms and greater improvements on the level of psychological capital at post-intervention than the control group that only received the usual care. To our knowledge, no studies have examined PCI in depression treatment-seeking samples. That was the central goal of the current research.

## Materials and Methods

### Participants

A total of 56 outpatients seeking treatment for depression were recruited from the First Hospital of Shanxi Medical University, the Third hospital of Shanxi Medical University and Shanxi Academy of Medical Sciences. Patients were eligible for the study if they present with clinically elevated symptoms of depression during the previous 2-week period and were aged between 18 and 65 years. To ensure that participants could complete the program safely and to apprehend of our findings, participants with (1) concurrent psychotherapy for depression during past 4 weeks; (2) a history of head injury, stroke, seizure, other central nervous system disease; (3) substance abuse; (4) a lifetime diagnosis of anxiety, bipolar disorder type I or II, schizophrenia; (5) strong suicidal ideation were excluded.

Diagnostic assessment was based on a structured diagnostic interview for DSM-IV criteria by the experienced clinical experts. Participants enrollment statistics and progress through the study are presented in Supplementary Figure S1. Of the 56 participants who were randomized to the PCI (*n* = 31) or control group (*n* = 25), two participants in the PCI discontinued treatment due to inconvenient to hospital following session 3. Thus, 54 participants (*n* = 29 in the PCI and *n* = 25 in the control group) were included in the study (see Supplementary Appendix B). Patients in each condition were equally likely to receive medication.

### Measurement

In addition to basic demographic characteristics (gender, marital status, family residence, education, and family history), the following two scales were assessed:

#### Depression

Depression were assessed using the Center for Epidemiological Studies Depression Scale (CES-D), which is a widely used 20-item self-report scale designed to measure depressive symptoms in a general population. Items are scored on a 4-point scale, with the total of CES-D score ranging between 0 and 60. A score of 16 points or more is generally considered suggestive of a depressive disorder, higher scores imply more severe depressive symptoms (Radloff, 1977). The coefficient alpha of the CES-D scale of the present study was 0.93.

#### Pychological Capital (PsyCap)

Positive PsyCap Questionnaire (PPQ) consists of 26 items on a 7-point scale to assess hope, self-efficacy, optimism and resilience (Zhang et al., 2010). It is a reliable and valid measure of PsyCap. In current study, the confirmatory factor analysis (CFA) of PsyCap showed a good fit to the data (χ^2/^*df* = 2.075, RMSEA = 0.065, TLI = 0.909, IFI = 0.926). The Cronbach’s alpha was 0.90. In terms of each subscale, the Cronbach’s alpha was 0.87 for hope, 0.91 for self-efficacy, 0.86 for optimism, 0.90 for resilience.

### Procedure

All subjects gave written informed consent prior to their participation in the study and the study was approved by the ethical review board of Shaoyang University. Participants who met the inclusion criteria and agreed to participate in the research were invited to complete the baseline evaluation. Following the baseline assessment, participants were randomly assigned to either the PCI or CAU group according to a random number generator.

Participants assigned to the PCI group completed four 45-minute weekly sessions of the PCI protocol (see Supplementary Appendix A). Prior to the first intervention, the researcher concentrated upon the introduction of each module and the general explanation of the effects of positive and negative emotions on depression. Following the last treatment session or approximately 4 weeks after the baseline, participants completed post-test sessions, which were identical to the pre-test. To establish the duration of treatment effects, participants in the PCI group completed the assessments 6-month following the post-test session.

Care as usual participants completed the pre-and post-assessment at a 4-week interval. Treatment were offered the PCI protocol after the study was terminated.

### Statistical Analyses

Statistical analyses were performed using SPSS version 18.0 in an intent-to-treat (ITT) basis (PCI = 29, CAU = 25), with *p* < 0.05 indicating statistical significance. Primarily outcome measures were analyzed descriptively. Analysis of covariance (ANCOVA) was used as a statistical technique to test group differences at post-treatment controlling for the pretreatment scores. Effect sizes including within-group and between- group controlled effect sizes were calculated to establish the magnitude of treatment response (Lakens, 2013).

## Results

### Preliminary Analyses

Demographic characteristics in both PCI group and CAU group at baseline were presented in Supplementary Appendix B. The two groups did not statistically differ in terms of gender, marital status, education, home residence, family history of depression, age (*ps* > 0.05).

### Main Treatment Effects

Table 1 displayed the details about the means, standard deviations and results of the ANCOVAs for the main outcomes. The results of ANCOVAs revealed that individuals in the PCI group demonstrated significantly greater PsyCap at post-treatment compared to participants in the CAU group. ANCOVA results also revealed that the PCI group reported experiencing significantly fewer symptoms of depression, at post-treatment relative to participants in the CAU group. As shown in Table 2, a significant treatment effect for the PCI group both within- and between-group was observed.

**TABLE 1:** Descriptive summaries of the treatment outcome measures for the PCI and CAU groups.

**Measure**	**Pre-test *M* (SD)**		**Post-test *M* (SD)**		**Results^a^ (Group)**		
	**PCI**	**CAU**	**PCI**	**CAU**	***F***	***p***	**η*^2^***
PsyCap	68.74 (11.93)	67.14 (7.26)	90.92 (8.66)	68.46 (7.19)	11.19	0.002	0.73
Hope	14.90 (3.81)	14.37 (3.56)	26.82 (4.24)	14.91 (3.28)			
Self-efficacy	20.70 (5.21)	19.33 (4.27)	28.91 (3.66)	20.61 (4.35)			
Optimism	14.02 (4.14)	14.28 (2.94)	24.75 (3.65)	14.63 (3.31)			
Resiliency	17.10 (6.24)	17.32 (5.46)	26.53 (2.43)	18.11 (4.92)			
Depression score					6.61	0.013	0.45
CES-D	29.90 (6.57)	29.63 (7.32)	14.61 (4.03)	20.60 (7.21)			

*Significant results after BH correction are presented in the table. PCI, psychological capital intervention; CAU, care as usual; PsyCap, psychological capital; CES-D, center for epidemiological studies depression scale. ^*a*^Analysis of covariance *(ANCOVA)* results comparing the PCI and CAU groups at post-assessment controlling for pre-assessment dependent outcomes.*

**TABLE 2:** Effect sizes for pre- to post-test changes in the PCI and CAU groups.

**Measure**	**Effect size**		
	**Within-group^a^**		**Between-group^b^**
	**PCI**	**CAU**	
PsyCap	2.16	0.39	2.86
Hope	2.98	0.23	3.09
Self-efficacy	1.67	0.29	1.82
Optimism	2.74	0.08	2.77
Resiliency	1.77	0.13	1.81
Depression score			
CES-D	–2.15	–0.41	–1.38

*PCI, psychological capital intervention; CAU, care as usual; PsyCap, psychological capital; CES-D, center for epidemiological studies depression scale. ^*a*^Within-group pre- to post-treatment effect sizes = [(post-treatment mean– pre-treatment mean)/(pretreatment standard deviation + post-treatment standard deviation)/2]. ^*b*^Between-group controlled effect sizes = (post-assessment PAI group covariance adjusted mean – post-assessment waitlist group covariance adjusted mean)/pooled standard deviation.*

### Maintenance of Treatment Effects

Table 3 depicted the means, standard deviations and results of the repeated measures ANOVAS in the PCI group at pre, post-test and 6-month follow-up. The repeated measures ANOVAs indicated significant main effects of Time. *Post hoc* test revealed that the main outcomes of significant difference from pre- to post-test and from pre- to 6-month follow-up for the PCI group (*ps* < 0.05), however, post-test and 6-month follow-up did not significantly differ (*ps* > 0.05).

**TABLE 3:** Descriptive summaries of the treatment outcome measures for the PCI group.

**Measure**	**Pre-test *M* (SD)**	**Post-test *M* (SD)**	**6-month follow-up *M* (SD)**	**Results^a^ (Group)**		
				***F***	***P***	**η*^2^_*p*_***
PsyCap	68.74 (11.93)	90.92 (8.66)	92.34 (9.28)	12.35	0.011	0.48
Hope	14.90 (3.81)	26.82 (4.24)	27.26 (5.42)			
Self-efficacy	20.70 (5.21)	28.91 (3.66)	29.62 (4.29)			
Optimism	14.02 (4.14)	24.75 (3.65)	25.03 (3.26)			
Resiliency	17.10 (6.24)	26.53 (2.43)	26.74 (4.77)			
Depression score				19.16	0.024	0.61
CES-D	29.90 (6.57)	14.61 (4.03)	14.52 (3.95)			

*Significant results after BH correction are presented in the table. PCI, psychological capital intervention; PsyCap, psychological capital; CES-D, center for epidemiological studies depression scale. ^*a*^Repeated measures analysis of variance (ANOVA) results for time in the PCI group.*

## Discussion

The findings of the present pilot study confirm that PCI resulted in significant significantly greater increases in PsyCap and decreases in depressive symptoms compared to a CAU group. Treatment effects were large in magnitude and persisted up to 6-months following termination of treatment. The current preliminary findings emphasis the potential value of targeting the positive resources in depression treatment directly, suggesting that PCI may be beneficial for individuals with impairing symptoms of depression. We took the first step in evaluating the efficacy of PCI for depression. Given that this is the first attempt to use the PCI for depression, our research extends the knowledge of treatment of depression and the application of PCI.

The PCI regimen shares much in common with other positive psychological interventions that focused on developing positive emotions as hope, gratitude, interest, pride, inspiration, love, joy and the establishment of strengths, skills, resilience and goals (Hobfoll, 2002; Seligman et al., 2005; Sin and Lyubomirsky, 2009; Dunn, 2012; Santos et al., 2013; Carr and Finnegan, 2014; Boumparis et al., 2016; Kwok et al., 2016). The principal differences between the PCI regimen and other similar programs is that combing the four components of PCI, these four resources compose a higherorder construct which is based on the commonalities these four first-order constructs share (Hobfoll, 2002; Luthans et al., 2007a; Avey et al., 2011; Newman et al., 2014; Nolzen, 2018). Integrating with the function of hope, self-efficacy, optimism and resilience, they might yield larger effect size than the sum of parts.

On the basis of these outcome data, we conclude that PCI has not only yield larger effects on PsyCap, but also on depression intervention. It is noteworthy that targeting the positive resources (PsyCap) resulted in reductions in depressive symptoms, changes that were comparable to those prevailing empirically establish interventions (McCarty et al., 2013; Carr and Finnegan, 2014; Reiter and Wilz, 2015; Kwok et al., 2016; Taylor et al., 2017). The results may be explained by multiple reasons from the perspective of positive psychology. One explanation might be that PCI teaches the individuals some strategies to develop the positive cognition, emotions and behaviors. These positive resources help the participants to deal with current emotion problems and enhance the capabilities to cope with future stress and adversities. Another explanation might be that individuals are born with the instinct to obtain happiness and the potential for continuous growth. Even individuals suffering from mental disorder such as depressive disorder have positive qualities and abilities which are inhibited compared with normal people. Through intervention regimen like PCI, the inhibited positive abilities developed so that they can recover from depression (Grawe and Grawe-Gerber, 1999; Flückiger and Wüsten, 2009; Reiter and Wilz, 2015). Hence, targeting the positive resource of depression is effective for depression treatment.

### Limitations

Strengths of this pilot study also include that we control the common method bias by adopting the self-report and structured interview and we used PCI for depression intervention which provide the new horizon for the treatment. The current findings should be interpreted in the context of several caveats. First, the efficacy of the PCI was evaluated in a small sample, which suggest limited power to detect statistically significant effects. Larger trials should be conducted to replicate and determine the stability of the effect. Second, another limitation of the current study may be the lack of correction for possible alpha inflation, however all tests were *a priori* planned comparisons and Bonferroni corrections with our small sample size would significantly reduce power. Third, outcomes were only examined at pre-, post-test and after 6-month. These results highlight the need for further research investigating multiple repeated assessments of the outcome and using longitudinal statistical models. In addition, wider recruitment, from other provinces and other ethnic would enhance the power of our data and the generalizability of our finding. These are issues that we are in the process of addressing in follow-up studies.

### Implications

This study is the first to investigate the effect of PCI on depression. The findings suggest that the intervention enhanced the levels of PsyCap, which in turn contribute to consequent decrease in depression symptoms. The current findings have several implications for clinical and public health in suggesting viable targets for the development of (1) education campaigns and health promotion and maintaining individual health or (2) treatment plans for depression or (3) other psychopathology.

Firstly, future public health messaging should emphasis how individuals using a variety of positive resources (e.g., PsyCap) as both prevention and management. Individuals who are not currently depressed but are interested in maintaining positive health could focus on building positive resources. That is, developing hope, self-efficacy, optimism and resilience.

Secondly, depression individuals benefit from developing positive resources (e.g., PCI) and over time can develop a suite of personalized options to be reliably used to prevent or manage depression symptoms.

Thirdly, the current findings also have promising application for a range of psychiatric conditions, including subsyndromal cases that fail to meet diagnostic criteria but may experience the functional impairment. The current intervention was tailored to depression, nevertheless, the PCI might be applied to other forms of psychopathology like anxiety, phobia or increasing the personals’ strengths, well-being, or other positive psychological resources.

## Conclusion

Results of the current pilot study provided initial support for the efficacy of PCI for depression treatment. The findings support the potential value of explicitly targeting the absence of positive resources in depression. Future research using more rigorous control conditions in larger samples is needed.

## Data Availability

The datasets for this manuscript are not publicly available because the data involved the subject’s privacy, the subjects didn’t agree to public. Requests to access the datasets should be directed to rjsong@sina.cn.

## Ethics Statement

This study was carried out in accordance with the recommendations of Shaoyang University with written informed consent from all subjects. All subjects gave written informed consent in accordance with the Declaration of Helsinki. The protocol was approved by the Shaoyang University.

## Author Contributions

RS, NS, and XS designed the study. RS analyzed the data and drafted the manuscript.

## Conflict of Interest Statement

The authors declare that the research was conducted in the absence of any commercial or financial relationships that could be construed as a potential conflict of interest.

## Supplementary Material

FIGURE S1 Descriptive summaries of the treatment outcome measures of PsyCap for the PCI group.

APPENDIX A Psychological Capital Intervention (PCI) Protocol.

APPENDIX B Patient Demographic Characteristics.
