# Supplementary figures and images for: The Efficacy of Psychological Capital Intervention (PCI) for Depression From the Perspective of Positive Psychology: A Pilot Study
Source: Front Psychol. 2019 Aug 7;10:1816. doi: 10.3389/fpsyg.2019.01816 (PMC6692487; doi:10.3389/fpsyg.2019.01816)

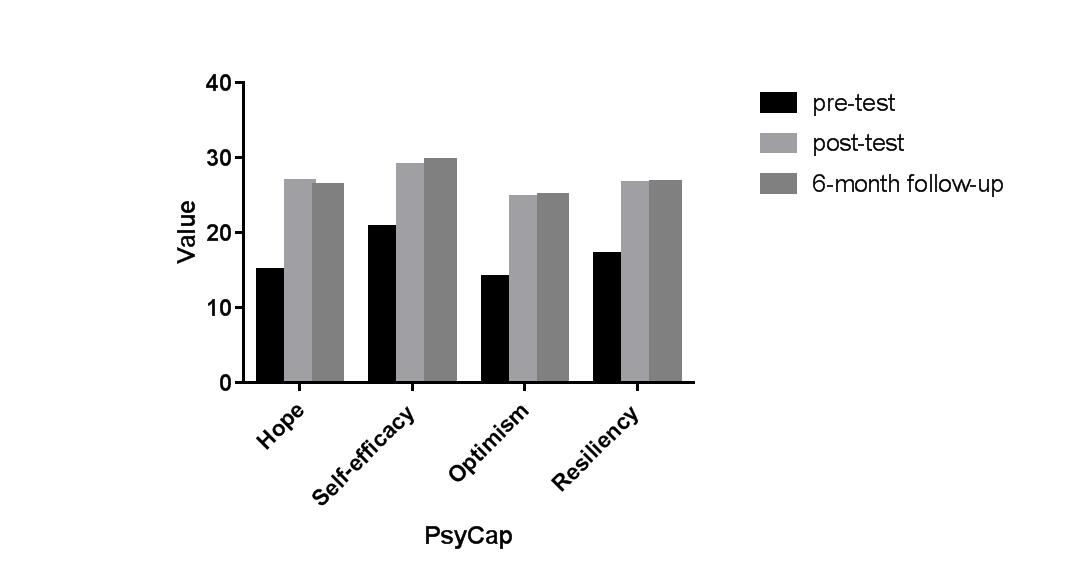

Supplement: FIGURE S1 — Descriptive summaries of the treatment outcome measures of PsyCap for the PCI group. [file Image_1.JPEG]
