# Supplementary material for: The Efficacy of Psychological Capital Intervention (PCI) for Depression From the Perspective of Positive Psychology: A Pilot Study
Source: Front Psychol. 2019 Aug 7;10:1816. doi: 10.3389/fpsyg.2019.01816 (PMC6692487; doi:10.3389/fpsyg.2019.01816)
Supplement: APPENDIX A — Psychological Capital Intervention (PCI) Protocol. [file Data_Sheet_1.doc]

Appendix A

***Psychological Capital Intervention (PCI) Protocol.***

| **Module** | **Process** |
| --- | --- |
| **Hope Development**  **(Session 1)** | - **Goal design** : identifying personally valuable goals that they will use throughout the 4 sessions, such goals include:    concrete end points to measure success   an approach framework which allows participants to positively move toward goal   identify sub-goals   - **Pathway generation:**    generate multiple pathways (brainstorm) to the goal mentioned above   consider the resources that each pathway required and identify the realistic pathway   - **Overcoming obstacles:**    consider the potential obstacles   discuss the strategy to overcome the obstacles |
| **Self-efficacy Development**  **(Session 2)** | - **Goal design：**Set earlier in the Hope Development - **Experience and model success and social persuasion and arousal:**    visualize accomplishing each step toward this goal   elicit positive emotions to and build the confidence to generate and implement plan to attain the goal |
| **Optimism Development**  **(Session 3)** | - **Self-efficacy training**: outlined in the session 2 - **Counteracting pessimism:**    ask the participant to state their failure and setback, then analyses the positive effect of the frustrate together   recall and experience the positive aspects of life |
| **Resiliency Development**  **(Session 4)** | - **Goal design:** Described in the Hope Development - **Enhancing asset factors:** discuss participants’ strengths, talents or skills to achieve the goal - **Avoiding risky or adverse events:** analyses the potential difficulties and discuss how to avoid them to realize the goal |

Appendix B

*Patient Demographic Characteristics.*

| Variable | PCI (n=29) | CAU (n=25) |
| --- | --- | --- |
| Gender, n (%) |  |  |
| Male | 12（41.3） | 9 （36.0） |
| Female | 17（58.7） | 16（64.0） |
| Marital status, n (%) |  |  |
| Single | 5 （17.2） | 3 （12.0） |
| Married | 23（79.3） | 21（84.0） |
| Divorced or widowed | 1 （3.5） | 1 （4.0） |
| Education, n (%) |  |  |
| Bachelor degree or above | 10（34.4） | 11（44.0） |
| Senior high school graduate or trade school | 10（34.4） | 8 （32.0） |
| Junior high school | 5（17.2） | 5 （20.0） |
| Primary school or below | 4（14.0） | 1 （4.0） |
| Family residence, n (%) |  |  |
| City | 13（44.8） | 11（44.0） |
| Town | 8（27.6） | 10（40.0） |
| Country | 8（27.6） | 4 （16.0） |
| Family history of depression, n (%) |  |  |
| Yes | 4（13.8） | 5 （20.0） |
| No | 25（82.8） | 20（80.0） |
| Age, mean (SD) | 41（12.3） | 42（13.2） |
